# Identification of an *in vivo* orally active dual-binding protein-protein interaction inhibitor targeting TNFα through combined *in silico/in vitro/in vivo* screening

**DOI:** 10.1038/s41598-017-03427-z

**Published:** 2017-06-13

**Authors:** Hadley Mouhsine, Hélène Guillemain, Gabriel Moreau, Najla Fourati, Chouki Zerrouki, Bruno Baron, Lucille Desallais, Patrick Gizzi, Nesrine Ben Nasr, Julie Perrier, Rojo Ratsimandresy, Jean-Louis Spadoni, Hervé Do, Patrick England, Matthieu Montes, Jean-François Zagury

**Affiliations:** 1 Laboratoire Génomique, Bioinformatique et Applications, EA 4627, Conservatoire National des Arts et Métiers, 2 rue Conté, 75003 Paris, France; 2 Peptinov SAS, Pépinière Cochin Santé, Hôpital Cochin, 29 rue du Faubourg Saint Jacques, 75014 Paris, France; 3 Laboratoire SATIE, CNRS, UMR 8029, Conservatoire National des Arts et Métiers, 292 rue Saint Martin, 75003 Paris, France; 4 Plate-forme de Biophysique des Macromolécules et de leurs Interactions, Proteopole Institut Pasteur, 25 rue du Dr Roux, 75015 Paris, France; 5 CNRS, UMR 7242, TechMedill, Bd Sebastien Brant, 67121 Illkirch, France

## Abstract

TNFα is a homotrimeric pro-inflammatory cytokine, whose direct targeting by protein biotherapies has been an undeniable success for the treatment of chronic inflammatory diseases. Despite many efforts, no orally active drug targeting TNFα has been identified so far. In the present work, we identified through combined *in silico*/*in vitro*/*in vivo* approaches a TNFα direct inhibitor, compound 1, displaying nanomolar and micromolar range bindings to TNFα. Compound 1 inhibits the binding of TNFα with both its receptors TNFRI and TNFRII. Compound 1 inhibits the TNFα induced apoptosis on L929 cells and the TNFα induced NF-κB activation in HEK cells. *In vivo*, oral administration of compound 1 displays a significant protection in a murine TNFα-dependent hepatic shock model. This work illustrates the ability of low-cost combined *in silico/in vitro/in vivo* screening approaches to identify orally available small-molecules targeting challenging protein-protein interactions such as homotrimeric TNFα.

## Introduction

Protein-protein interactions (PPI) represent a large class of therapeutic targets that play a crucial role in biological processes. Despite their importance, they were considered intractable due to their large and flat topology compared to classical small molecule binding sites^1^. Considerable progress was achieved in the last decade since 27 PPIs have now been tackled by small molecules^2, 3^ including organometallic compounds^4–6^ and dendrimers^7^. PPIs are still considered to be a very challenging class of targets for therapeutic applications^8, 9^. Historically, PPI inhibitors are larger and more hydrophobic than “drug-like” orally available compounds^10^. Despite their “excessive” logP or molecular weight, some PPI inhibitors such as navitoclax^11^ (molecular weight 975 g.mol^−1^) or venetoclax^12^ (molecular weight 868 g.mol^−1^) are orally available. Among the different strategies devised to inhibit PPIs, directed allosteric modulation could provide a potential way forward for the most difficult targets^13^.

Tumor Necrosis Factor alpha (TNFα) is a homotrimeric cytokine of the immune system whose overproduction has been associated with several chronic inflammatory diseases such as rheumatoid arthritis, Crohn’s disease or psoriasis^14^. Clinically approved inhibitors of TNFα include monoclonal antibodies (infliximab, adalimumab) and soluble receptors of TNFα (etanercept). These biotherapies display several drawbacks including opportunistic infections^15^ and treatment resistance due to autoimmune reactions^16^ that could be addressed by small molecule modulators.

Identified in 2005, SPD304 constitutes a reference allosteric modulator of TNFα that inhibits its activity by disrupting TNFα homotrimeric form^17^. SPD304 cannot be used *in vivo* due to his high toxicity^18^. Despite many efforts^5, 18–27^ no orally available TNFα inhibitor has been identified so far.

In the present work, in order to identify allosteric modulators of TNFα, we targeted the binding site of SPD304 with a large compound collection through the use of *in silico, in vitro* and *in vivo* screening. We report the structure and properties of our best confirmed hit, compound 1, a high affinity small molecule inhibitor of TNFα that inhibits the activity of TNFα *in vitro* and is orally active *in vivo* in a reference TNFα-dependent murine model^27–29^. This work illustrates the ability of current virtual screening methods to identify high affinity orally available compounds targeting challenging PPIs such as TNFα.

## Results and Discussion

**Figure 1:**
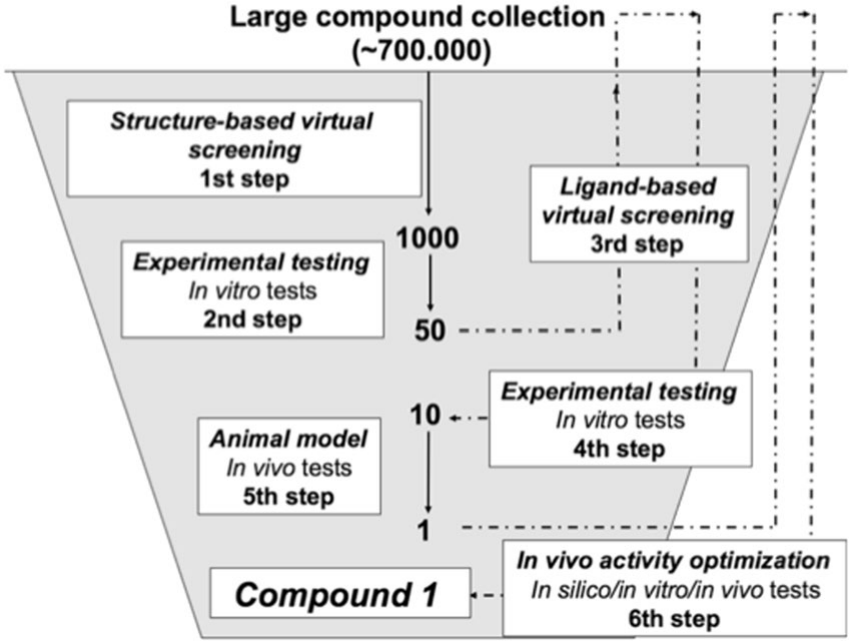
Flowchart of the screening protocol used in the study. *1st step*. A collection of 700,000 drug-like commercially available compounds was screened *in silico*. Molecular docking was performed using Surflex-dock version 2.5. After visual inspection, a 1000 compound hit list was selected for experimental testing. *2* ^*nd*^ *step*: The inhibitory activity of the compounds composing the hit list was evaluated *in vitro* on human TNFα induced apoptosis on the L929 cell line. Top hit compounds displayed an IC_50_ between 1 and 100 µM. *3* ^*rd*^ *step*: 2D/3D similarity search methods were used to identify analogues of the top hits identified after step 2. Up to 100 analogues were found per top hit with a Tanimoto similarity score >0.6. *4* ^*th*^ *step*: As in step 2, their inhibitory activity was evaluated *in vitro* on human TNFα induced apoptosis on the L929 cell line. The 10 best compounds after these 4 steps were selected as candidates for *in vivo* evaluation on a murine model. *5* ^*th*^ *step*: The *in vivo* evaluation of the candidates was performed in the TNFα-dependent hepatic shock model triggered with LPS/D-Galactosamine via force-feeding. After this step, 1 *in vivo* active compound was selected. *6* ^*th*^ *Step*: Using 2D/3D similarity search methods, we searched in our large compound collection for new analogues of this best compound identified after step 5. Up to 500 analogues were identified and purchased from the chemical supplier. As in step 2, their inhibitory activity was evaluated *in vitro* on human and murine TNFα. The 9 best compounds were evaluated *in vivo* in our murine hepatic shock assay by force-feeding as described in step 5. The best compound identified after the 6^th^ step is compound 1.

**Figure 2:**
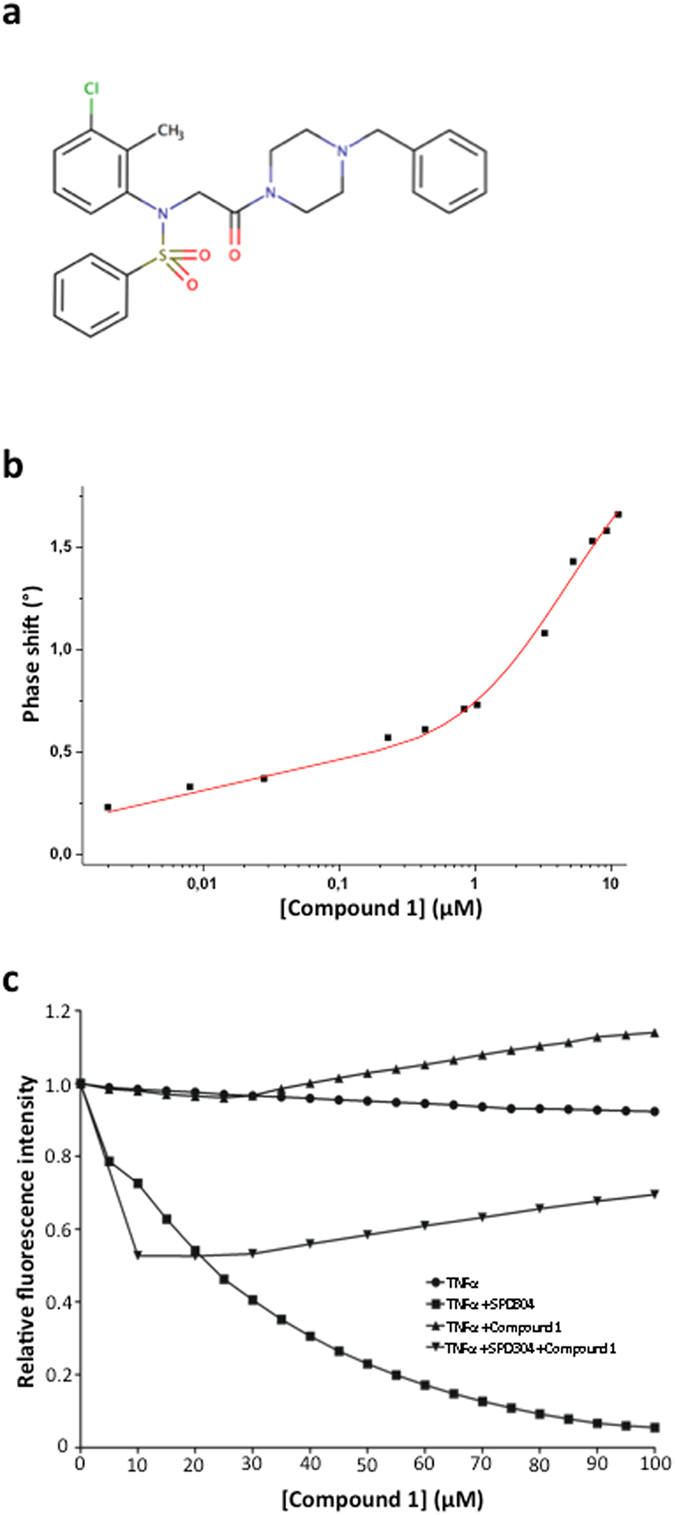
(**a**) Structure of compound 1. (**b**) Dissociation constants of TNFα/Compound 1. Determination of dissociation constants of TNFα/compound 1 complex, from gravimetric biosensor response, by using a “two-site binding” model. *K*_*d1*_ = 4.79 ± 1.12 µM and *K*_*d2*_ = 2.31 ± 1.03 nM. (**c**) Intrinsic Tryptophane Fluorescence. Intrinsic Tryptophan Fluorescence of 0.5 μM TNFα diluted in Phosphate Buffered Saline in the presence of DMSO alone, SPD304 (5–100 µM) in DMSO, compound 1 (5–100 µM) in DMSO or SPD304 (25 µM) + compound 1 (10–100 µM) in DMSO.

In order to identify allosteric modulators of TNFα, we carried out a hierarchical *in silico* and *in vitro* screening of the top 0.2% scoring compounds of a collection of 700,000 commercially available compounds by targeting the binding pocket of SPD304 in TNFα identified by He *et al*.^17^ (Supplementary Fig. S1). This hierarchical protocol, described in Fig. 1a, led to the identification of compound 1, whose structure is depicted in Fig. 2a, and whose analytics are presented in Supplementary Fig. S2a–d.

### Dissociation constants and Ligand efficiency of compound 1 with TNFα

Dissociation constants of the TNFα/SPD304 and TNFα/compound 1 complexes were determined from gravimetric measurements using a Surface Acoustic Wave (SAW) biosensor^30–32^. On the TNFα/SPD304 complex, the phase shift variations (ΔΦ) suggested a “one-site binding” with a corresponding *K*_*d*_ constant of 9.1 ± 1.1 µM which is consistent with the Kd values obtained by Papaneophytou *et al*. in the literature using a fluorescence binding assay (*K*_*d*_ = 5.4 ± 0.2 μM)^33^. On the TNFα/compound 1 complex, the phase shift variations (ΔΦ) suggested a “two-site binding” (Fig. 2b). The corresponding equilibrium dissociation constants *K*_*d*_ were respectively in the micromolar range (4.79 ± 1.12 µM) and in the nanomolar range (2.31 ± 1.03 nM) at room temperature. The corresponding ligand efficiencies of compound 1 (LE_1_ = 0.22 and LE_2_ = 0.37) are in the range of most of the protein-protein interaction inhibitors^9^.

### Modification of the intrinsic tryptophan fluorescence profile of TNFα by compound 1

The intrinsic tryptophan fluorescence (ITF) of TNFα is modified by adding compound 1 in a dose dependent manner at the 20–100 µM range (Fig. 2c). The modification of the ITF profile by compound 1 is different to the one obtained with SPD304^17^ which is consistent with a mid micromolar affinity binding of compound 1 in an additional binding pocket at the surface of the homotrimer close to tryptophan residues.

### Predicted binding modes of compound 1 on TNFα

**Figure 3:**
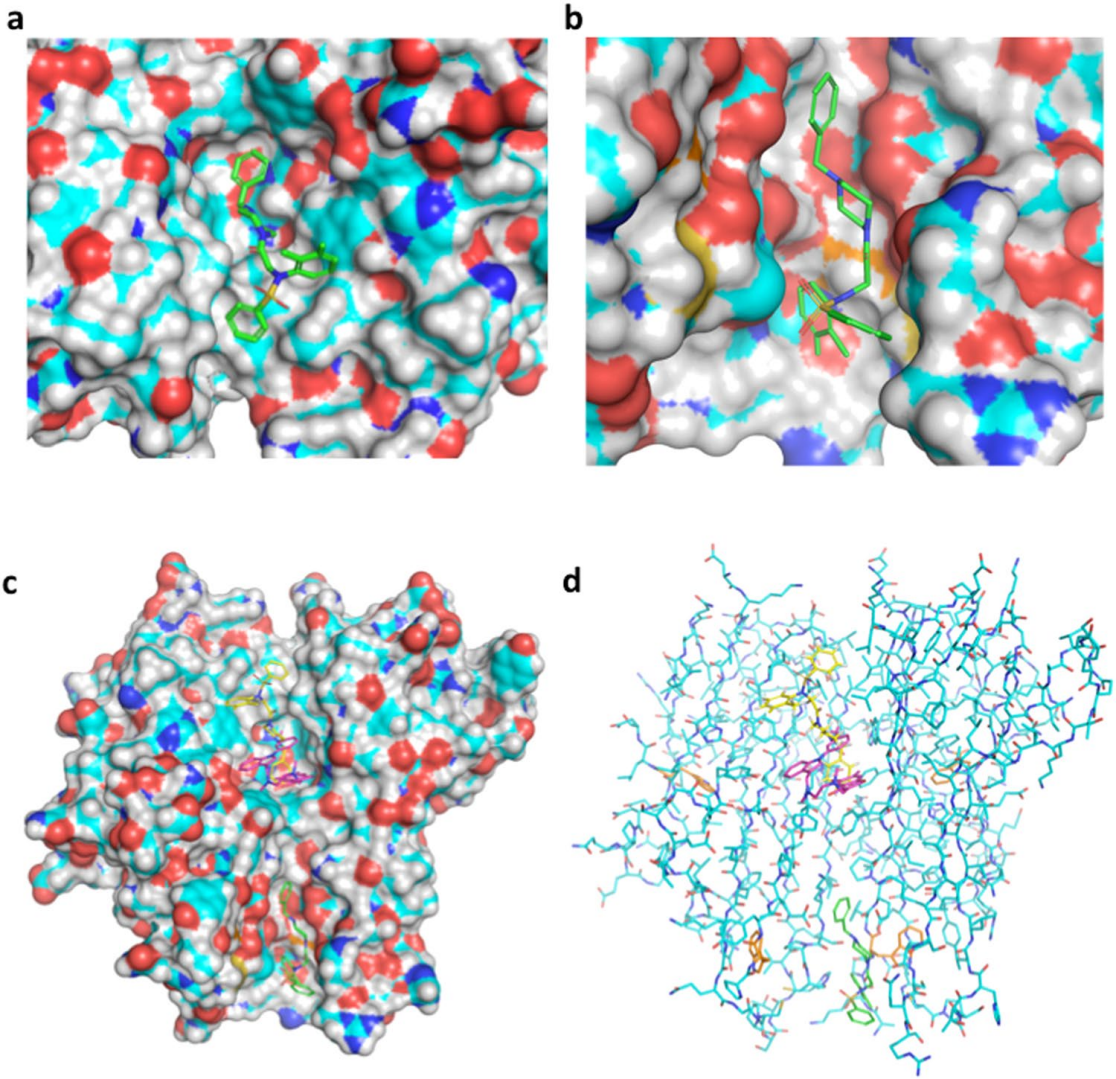
(**a**) Illustration of the top scoring binding mode of compound 1 with Surflex-dock in the TNFα binding site defined for the study (PDBID:2AZ5). (**b**) Alternate binding mode of compound 1 (green) predicted using Surflex-dock on the surface of TNFα dimer. Tryptophan residues are displayed in orange. (**c**) Secondary pocket on the structure of human dimeric TNFα. (**d**) Dual binding of compound 1 (yellow and green) predicted with Surflex-dock on the surface of the TNFα dimer co-cristallized with SPD304 displayed in purple (PDB ID: 2AZ5). Tryptophan residues are displayed in orange.

The top-scoring binding mode of compound 1 predicted using Surflex-dock^34^ is illustrated in Fig. 3a. As expected from the highly hydrophobic surface of the binding pocket of SPD304^17, 23^, compound 1 displays numerous hydrophobic interactions with TNFα, its benzyl-piperazine moiety being deeply inserted in the primary binding pocket of SPD304 surrounded by Y232, Y264, Y179, L239 and L177. The phenyl-sulfonamide moiety of compound 1 displays additional hydrophobic interactions with an extension of the SPD304 binding pocket surrounded by L129, L43, L270, I268 and I127. This binding mode of compound 1, compared to the binding mode of SPD304, would not only hinder the positioning of the side chain of Y119 of the disrupted TNFα monomer^17^ but also the side chains of L57, L157 and V123 of the disrupted TNFα monomer.

To illustrate the alternate binding mode of compound 1 on TNFα, we explored the whole surface of the structure of human trimeric TNFα (PDB ID: 1TNF) to identify an additional binding pocket at the surface of the homotrimer that contains tryptophan residues (Fig. 3b). After a careful visual inspection, a pocket has been identified at the junction of the three TNFα subunits that is conserved in the homodimeric structure cocristallized with SPD304 (PDB ID: 2AZ5). This secondary pocket illustrated in Fig. 3c could be the one we suspected after gravimetric studies (dual binding) and intrinsic tryptophan fluorescence measurements (alternate modification of the ITF profile) since it is the only one on the surface of TNFα that contains tryptophan residues (it contains one tryptophan residues per TNFα monomer). The top scoring binding mode of compound 1 on this new binding pocket on the surface of the TNFα dimer predicted using Surflex-dock is illustrated in Fig. 3d.

### Activity of compound 1 on TNFα-TNFRI and TNFα-TNFRII interactions

**Figure 4:**
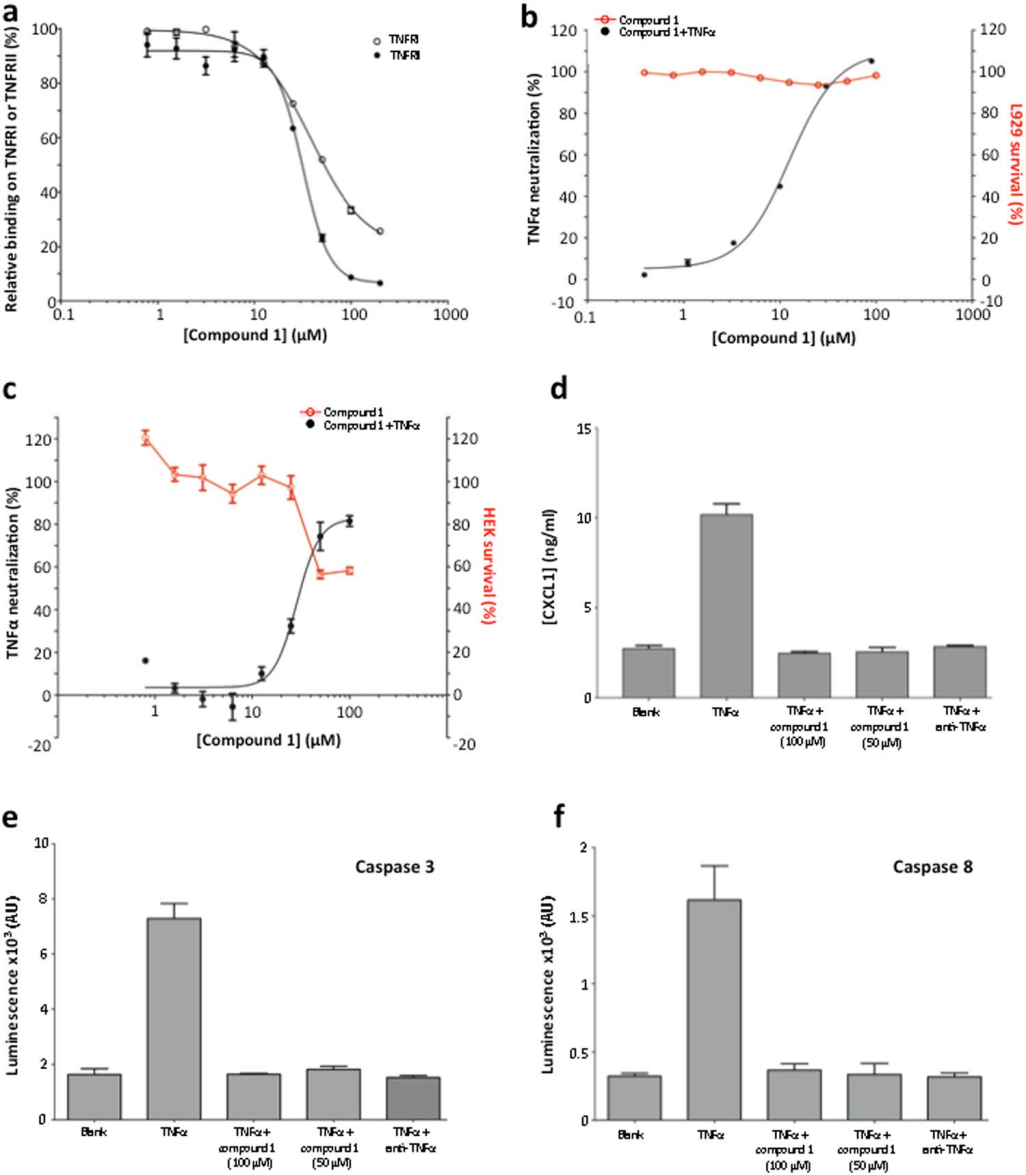
(**a**) Relative binding on TNFRI and TNFRII. Compound 1 inhibits the interaction between TNFα and its receptors TNFRI and TNFRII in a dose dependant manner. TNFRI IC_50_ = 37 μM, TNFRII IC_50_ = 31 μM. (**b**) Neutralization of TNFα and survival of L929 cells. Compound 1 inhibition of TNFα induced apoptosis in L929 cell line. Data represent neutralization of TNFα in presence of various concentrations of compound 1. IC_50_ = 12 μM (**c**) Neutralization of TNFα and survival of HEK cells. Compound 1 inhibition of the TNFα signaling pathway on HEK cells transfected with a reporter gene under the control of NF-κB. Data represent the neutralization of TNFα in the presence of different concentrations of compound 1. IC_50_ = 10 μM. (**d**) Inhibition of CXCL1 secretion in L929 supernatants in presence of various concentrations of compound 1 after stimulation with TNFα (5 ng/ml). (**e**) Inhibition of caspase 3 activity in presence of compound 1. (**f**) Inhibition of caspase 8 activity in presence of compound 1.

We evaluated the inhibitory activity of compound 1 on the binding of TNFα to its receptors TNFRI and TNFRII using an ELISA assay. Compound 1 displayed IC_50_ of 37 µM on TNFα/TNFRI and 31 µM on TNFα/TNFRII (Fig. 4a). These values are comparable to the IC_50_ of 22 µM obtained with SPD304 on TNFα/TNFRI^17^.

### Activity of compound 1 on TNFα functional cellular models

Compound 1 inhibited the induction of apoptosis by TNFα in the L929 cell line with an IC_50_ of 12 µM (Fig. 4b and Supplementary Movie). In the same assay, SPD304 displayed a high cellular toxicity (no cells survived with more than 30 µM of SPD304). The cellular activity of compound 1 was confirmed on the TNFα signaling pathway, by using a HEK cell line expressing TNFRI and transfected with a reporter gene under the control of five NF-κb binding sites, where compound 1 displayed an IC_50_ of 10 µM (Fig. 4c). On the same assay, SPD304 displayed a high cellular toxicity (no cells survived with more than 10 µM of SPD304). Since TNFα and TNFβ share 30% sequence identity, the activity of compound 1 on TNFβ in the L929 cell line was also evaluated. Compound 1 inhibited the induction of apoptosis by TNFβ with an IC_50_ of 28 μM and a maximal survival of 40% at 100 μM (Supplementary Fig. S3).

### Activity of compound 1 on CXCL1 expression, caspases and protein kinases related to the TNFα pathway

Compound 1 was tested for its ability to inhibit the production of CXCL1 on L929 cells since is triggered by TNFα. The supernatants from treated L929 cells with TNFα and compound 1 contained significantly less CXCL1 than the supernatants from untreated L929 cells as depicted in Fig. 4d. Compound 1 inhibited the secretion of CXCL1 at a similar level to the anti-TNFα antibody. We evaluated the ability of compound 1 to inhibit the activation of caspase 3 and caspase 8, widely used as readouts for TNFα signaling. Compound 1 blocked the activation of caspase 3 and caspase 8 (Fig. 4e,f), in a dose dependent manner. Compound 1 was tested for direct inhibition of caspases and kinases activities related to the TNFα pathway (IκB kinases (IKK), JNK1, p38 and Syk, caspase 3, caspase 8). Compound 1 displayed no significant effect on these proteins at 1 µM (Supplementary Fig. S4).

### Activity of compound 1 on murine *TNFα*

Compound 1 inhibited both human and murine TNFα with similar IC_50_s on the TNFα-induced apoptosis assay on L929 cells (~10 µM).

### *In vivo* activity of compound 1

**Figure 5:**
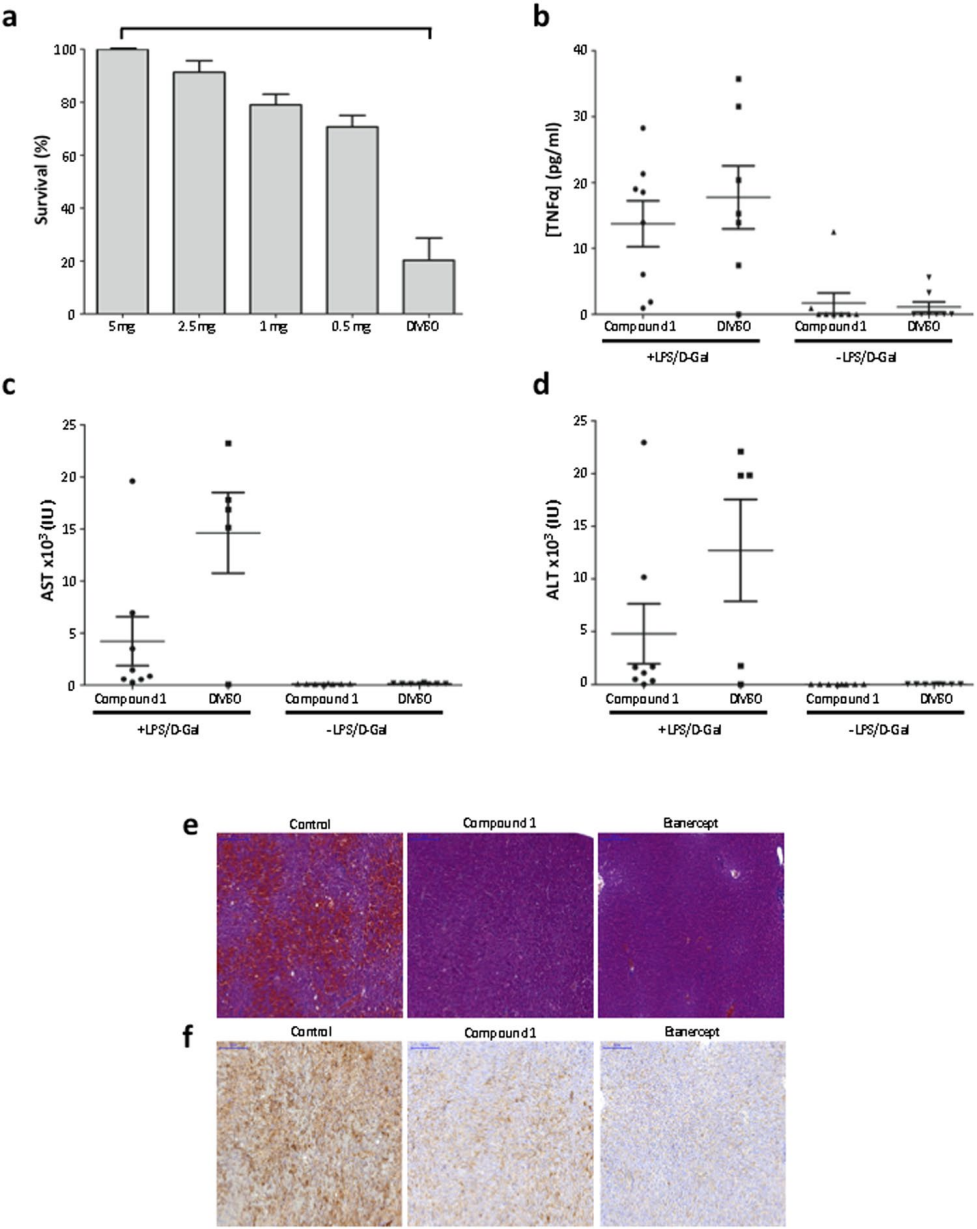
Effect of compound 1 in an *in vivo* murine model of LPS/D-Galactosamine induced shock. (**a**) Mice survival after force-feeding with different doses of compound 1 and an intraperitoneal injection of LPS/D-Galactosamine. Groups of eight mice were used. Values are mean ± s.e.m. (**b**) TNFα levels in mice sera three hours after the induction of the shock by LPS/D-Galactosamine. Mice were forced-fed with 5 mg compound 1 in DMSO. Groups of eight mice were used. Values are mean ± s.e.m. (**c**) Aspartate aminotransferase (AST) levels in mice sera eight hours after the induction of the shock. Mice were forced-fed with 5 mg compound 1 in DMSO. Values are mean ± s.e.m. (**d**) Alanine aminotransferase (ALT) levels in mice sera eight hours after the induction of the shock. Mice were forced-fed with 5 mg compound 1 in DMSO. Values are mean ± s.e.m. (**e**) Livers sections (H&E staining) from mice forced-fed with DMSO (control) or compound 1 or injected with etanercept and an intraperitoneal injection of LPS/D-Galactosamine. (**f**) Liver sections from mice forced-fed with DMSO (control) or compound 1 or injected with etanercept and an intraperitoneal injection of LPS/D-Galactosamine. Sections were incubated with an anti-cleaved caspase-3 antibody and revealed with an anti-rabbit antibody and DAB.

The *in vivo* activity of compound 1 was evaluated in a TNFα dependent murine model, the LPS/D-Galactosamine induced shock assay^29^. As shown in Fig. 5a, compound 1 exhibited a fully protective effect with an oral administration of 5 mg of compound 1 per mouse eight hours before the induction of the shock (p < 4.10^−3^). The oral administration of 5 mg of compound 1 did not affect the mice serum level of TNFα induced after the shock (Fig. 5b) but diminished the mice serum level of AST/ALT which transcribes the damage induced to hepatocytes (Fig. 5c,d). We conducted hispathological and immunohistochemical analyses on the livers of the LPS/D-Galactosamine injected mice, treated with the vehicle (DMSO), compound 1 or etanercept (Fig. 5e,f). The mice that received only the vehicle displayed important liver damage and hemorrhage whereas the mice treated with compound 1 or etanercept (Fig. 5e) displayed reduced liver damage and hemorrhage. The immunohistochemical analysis of the mice livers treated with compound 1 or etanercept displayed a reduced expression of cleaved caspase 3 (Fig. 5f), which is consistent with a reduction of the liver apoptosis in these animals. Taken together, these results confirmed that compound 1 did not affect the production of TNFα but inhibited TNFα induced damage on the mice liver by preventing caspase 3 induced apoptosis of hepatocytes.

## Conclusion

Using an *in silico*/*in vitro*/*in vivo* screening approach, we have identified compound 1 that binds TNFα with high affinity and inhibits its activity *in vitro* and *in vivo* via oral administration. Compound 1 physicochemical profile displays some drawbacks that could be addressed after a medicinal chemistry program, in particular a moderate solubility in aqueous solvents and a low bioavailability rendering necessary administration of several milligrams of compound per mouse. However, we could demonstrate that despite these drawbacks, compound 1 displayed a fully protective effect on an acute TNFα dependent mouse model in which large quantities of TNFα are released and inhibited by the compound. Compound 1 displayed a reduced inhibiting activity on TNFβ compared to TNFα. Targeting both TNFα and TNFβ could be beneficial for the treatment of rheumatoid arthritis^35^ since etanercept, a treatment of reference, displayed effective downregulation of both TNFα and TNFβ^36, 37^.

The next steps for the development of a drug based on the properties of compound 1 will be to improve its physicochemical profile, assess its full pharmacodynamics properties and evaluate its protective effect in a TNFα dependent chronic animal model closer to the physiopathology of chronic inflammatory diseases. This work illustrates a first step in the identification of orally available small molecule cytokine inhibitors that could be the basis for the development of alternate strategies to the biologics used for the treatment of chronic inflammatory diseases. In addition, this work highlights the ability of low-cost integrative *in silico/in vitro/in vivo* screening approaches to identify small-molecules targeting challenging protein-protein interactions such as homotrimeric TNFα.

## Experimental Procedures

### Materials, cell line and mice

Compounds were obtained from Chembridge (San Diego, CA, USA). Dimethyl Sulfoxide (DMSO), Lipopolysaccharide (LPS), TMB, Thiazolyl Blue Tetrazolium Bromide (MTT) and DAB were obtained from Sigma-Aldrich (Saint Quentin Fallavier, France). Human TNFα, human TNFβ and anti-TNFα antibody were obtained from R&D Systems (Lille, France). Anti-cleaved caspase 3 was obtained from Cell Signaling Technology (St Quentin en Yvelines, France), Anti-rabbit antibody coupled with HRP was obtained from Abcam (Paris, France) Dulbecco’s Modified Eagle and Phosphate Buffered Saline (PBS) were obtained from Pan Biotech (Brumath, France). Actinomycin D and D-Galactosamine were obtained from Fisher (Illkirch, France). L929 cell line has been grown in the Laboratory for years. HEK-Blue™ TNFα reporter cell line and QUANTI-Blue™ were obtained from InvivoGen (Toulouse, France). 7 weeks-old female Balb/C mice were obtained from Charles River Laboratories (L’Arbresle, France). Mice used in all experiments were handled according to the guidelines and protocols were approved by the ethical commitee of Paris Descartes University, France.

### *In silico* screening

#### Structure preparation

The binding site has been defined at 4 Å around the co-crystallized SPD304 ligand in the structure of the TNFα dimer (PDB id: 2AZ5, Supplementary Fig. 1). Hydrogen atoms were added using Chimera^38^.

#### Compound collection

The 900,000 compounds Chembridge screening compound collection was retrieved from www.hit2lead.com. After an ADME-tox filtering using FAF-drugs2^39^, 700,000 compounds were selected to constitute our commercially available drug-like compound collection.

#### Structure-based virtual screening

Molecular docking was performed using Surflex-dock version 2.5^34^. Surflex-dock is based on a modified Hammerhead fragmentation/reconstruction algorithm to dock compounds flexibly into the binding site. The query molecule is decomposed into rigid fragments that are superimposed to the Surflex-protomol *i.e*., molecular fragments covering the entire binding site. The docking poses are evaluated by an empirical scoring function. The protomol generation step has been performed using the options proto_thresh = 0.5 and proto_bloat = 4. Screening step was performed using the option–pscreen.

#### Ligand-based virtual screening

Ligand-based virtual screening was performed using the 2D/3D similarity search methods implemented in the webservice provided by www.hit2lead.com. Analogues identifications were performed using 2D and 3D similarity search with a 60% Tanimoto-based 2D and 3D similarity cut-off. Compounds were selected for experimental tests after a careful visual inspection of the retrieved analogues.

### Gravimetric measurements for the determination of dissociation constants

Gravimetric measurements were performed using surface acoustic wave (SAW) sensors. TNFα solutions were prepared by diluting 10 µg of lyophilized TNFα in 1 ml of PBS. They were kept in the freezer (at −18 °C) and put at room temperature just before their further grafting onto the gold surface area of the SAW sensor. We prepared stock solutions of 0.5 g/l of SPD304 in PBS and 0.1 g/l of compound 1 in HCl 0.1 M/H_2_O (1/1: v/v). pH was adjusted to ≈7 before the injection of the analyte of interest into the microfluidic channel of the detection system.

Phase shift variations (ΔΦ) versus compound concentration (C) were fitted using equation 1

$${\rm{\Delta }}{\rm{\Phi }}=\frac{{{\rm{\Phi }}}_{{\rm{s}}{\rm{a}}{\rm{t}}}\times {\rm{C}}}{{\rm{K}}{\rm{d}}+{\rm{C}}}$$

where Φ_sat_ represents the maximum sensor’s response to the compound binding and Kd represents the equilibrium dissociation constant.

In the case of a two-site binding, equation 2 was used for fitting:

2 $${\rm{\Delta }}{\rm{\Phi }}=\frac{{{\rm{\Phi }}}_{{\rm{s}}{\rm{a}}{\rm{t}},1}\times {\rm{C}}}{{{\rm{K}}}_{{{\rm{d}}}_{1}}+{\rm{C}}}+\frac{{{\rm{\Phi }}}_{{\rm{s}}{\rm{a}}{\rm{t}},2}\times {\rm{C}}}{{{\rm{K}}}_{{{\rm{d}}}_{2}}+{\rm{C}}}$$

where Φ_sat1_ and Φ_sat2_ are related to the maximum sensor’s response for each binding site and K_d1_ and K_d2_ are their respective dissociation constants.

### Measurement of TNFα intrinsic fluorescence

All samples were brought to 10 mM phosphate buffer, 0.05% Tween20, 1% DMSO. Fluorescence readings were made with a Quantamaster QM4CW spectrofluorimeter (Photon Technology International), by exciting TNFα at 290 nm and measuring the emission peaks at 322 nm. Compound 1 inner filter effects were corrected using absorbance measurements of compound alone solutions at 290 nm and 322 nm.

### TNFα-TNFRI and TNFα-TNFRII binding ELISA

Microtiter plates were coated with 12.5 ng of TNFRI or TNFRII in 125 μl of PBS per well at 37 °C. The wells were washed, blocked with PBS/BSA 2% for three hours and washed as before. Serial dilutions of compounds were mixed with a fixed quantity of TNFα in PBS/BSA 1% and incubated two hours at 37 °C. 125 μl of the mix were added to the wells and plates were incubated overnight at 4 °C. Wells were washed incubated with 37.5 ng of TNFα biotinylated antibody in 125 μl of PBS/BSA 1% for two hours at 37 °C. Wells were washed and incubated with avidin-HRP (1:500) in 125 μl of PBS/BSA 1% for 30 minutes at 37 °C, 5% CO_2_. After washing, TMB solution was added to wells, quenched with 63 μl of 1 M H_2_SO_4_ solution. Absorbance was measured at 450 nm.

### Neutralization of cellular TNFα induced apoptosis

80% confluent L929 cells were plated in flat bottom plates at 4 × 10^5^ cells per well in 100 μl of DMEM medium containing 10% FBS, 2 mM L-Glutamine, 100 U/ml Penicillin − 100 μg/ml Streptomycin and incubated for one night at 37 °C, 5% CO_2_. TNFα or TNFβ (150 pg/ml), Actinomycin D (4 μg/ml) and the compounds at different concentrations (ranging from 100 μM to 0.8 μM) were mixed in 150 μl DMEM medium 1% FBS, 2 mM L-Glutamine, 100 U/ml Penicillin−100 μg/ml Streptomycin in U-bottom plates. After two hours incubation at 37 °C, 5% CO_2_, 100 μl of the mix was added to the plated cells and incubated at 37 °C, 5% CO_2_ for 24 hours. Supernatants were discarded and 100 μl of MTT at 0.5 mg/ml were added to wells. After two hours, supernatants were discarded and 200 μl of DMSO were then added. Plates were read at 570 nm with a spectrophotometer providing the optical density (OD) of each well. The percentage of neutralization of TNFα by a compound was calculated using equation 3:

3 $$ \% Neutra=\frac{ODcompound-ODTNF\alpha }{ODcells-ODTNF\alpha }\times 100$$

An IC_50_ could be computed from the percentage of neutralization for each compound.

### Secreted embryonic alcaline phosphatase reporter assay

80% confluent HEK-Blue™ TNFα were plated in flat bottom plates at 5 × 10^4^ per well in 100 μl of DMEM containing 2 mM L-Glutamine, 100 U/ml Penicillin − 100 μg/ml Streptomycin and incubated at 37 °C, 5% CO_2_. Serial dilutions of compounds (ranging from 100 μM to 0.8 μM) were mixed with 400 pg/ml of human TNFα in DMEM containing 2% of FBS, 2 mM L-Glutamine, 100 U/ml Penicillin−100 μg/ml Streptomycin in U-bottom plates. After two hours of incubation at 37 °C, 5% CO_2_, 100 μl of the mix was added to the plated cells and incubated 24 hours at 37 °C, 5% CO_2_. 20 μl of supernatants were incubated for 3 hours with 180 μl of QUANTI-Blue™ to reveal secretion of phosphatase alcaline and plates were read at 620 nm with a spectrophotometer providing the optical density (OD).

### Activation of Caspases 3 and 8 assays

Caspases 3 and 8 activation was determined using Caspase-Glo^®^ 3 and Caspase-Glo-8^®^ assays kits (Promega) according to the manufacter’s procedures. After 8 hours of treatment, plates were equilibrated at room temperature. 100 μl Caspase-Glo 3^®^ and Caspase-Glo 8^®^ reagent were added to each well. Luminescence was read on a FLUOstar OPTIMA reader.

### Measurement of the concentration of CXCL1

Cells were plated at 2.5 × 10^5^ cells/well in 24-well plates and treated as indicated. Eight hours later, supernatants were recovered and kept at −20 °C for later use. The concentration of murine CXCL1 was measured in supernatants with a dosage ELISA kit obtained from R&D Systems (Lille, France) according to the manufacter’s procedures.

### Inhibition activity of kinases and caspases related to TNFα pathway

Kinases and caspases activities in presence of 1 µM of compound 1 were carried out by CEREP/Eurofins (Celle-Lévescault). The activity of compound 1 on the different kinases and caspases was compared to control values. Data are expressed as percentages of inhibition of control values.

### LPS/D-Galactosamine induced lethal shock

7 weeks-old Balb/C mice were force-fed with 100 μl of a solution of DMSO containing various doses of compound 1 (5 mg down to 0.5 mg) eight hours before receiving an intraperitoneal injection of 200 μl of PBS containing 0.1 μg of LPS and 20 mg of D-Galactosamine. A control group (DMSO) was force-fed with 100 μl of a solution of DMSO alone eight hours before to be injected with 200 μl of LPS/D-Galactosamine solution. Mice survival was monitored for 48 hours after the injection of LPS/D-Galactosamine.

#### Histological and immunohistochemical studies of livers

7 weeks-old Balb/C mice were forced-fed with a solution of DMSO containing 5 mg of compound 1 eight hours before receiving an intraperitoneal injection of 200 μl of PBS containing 0.1 μg of LPS and 20 mg of D-Galactosamine. Eight hours after LPS/D-Galactosamine injection, mice were sacrificed by cervical dislocation and livers were harvested, fixed in 4% formalin and embedded in paraffin. Serial 4 μm sections were stained with hematoxylin and eosin or incubated with an anti-cleaved caspase 3 antibody (1:300) and revealed with an anti-rabbit antibody and DAB. Slides were scanned with a slide scanner (Perkin Elmer) and analyzed with Pannoramic viewer (3D HISTECH).

### Cytokines quantification

7 weeks-old Balb/C mice were forced-fed with a solution of DMSO containing 5 mg of compound 1 eight hours before receiving an intraperitoneal injection of 200 μl of PBS containing 0.1 μg of LPS and 20 mg of D-Galactosamine. Three hours after LPS/D-Galactosamine injection, mice were anesthetized with ketamine/xylazine to recover blood by cardiac puncture. Sera were kept at −80 °C until assay.

TNFα in serum was quantified using U-CyTech biosciences kits (Utrecht, The Netherlands) according to manufacturer’s procedures.

### AST/ALT quantification

7 weeks-old Balb/C mice were forced-fed with a solution of DMSO containing 5 mg of compound 1 eight hours before receiving an intraperitoneal injection of 200 μl of PBS containing 0.1 μg of LPS and 20 mg of D-Galactosamine. Eight hours after, mice were anesthetized by an injection of ketamine/xylazine to recover blood by cardiac puncture. Sera were kept at −80 °C until assay. Quantification of AST/ALT was performed using Architec ci 8200 analyser (Abbott) with reagent kits from Abbott.

### Ethics

All study protocols and experimental procedures were approved by the Paris Descartes University ethical comittee and were carried out in accordance with the approved guidelines.

### Statistics

One-tailed p-values were calculated using Fisher’s exact test.

## Electronic supplementary material

Supplementary Information

Supplementary Movie
