# Supplementary material for: Identification of an in vivo orally active dual-binding protein-protein interaction inhibitor targeting TNFα through combined in silico/in vitro/in vivo screening
Source: Sci Rep. 2017 Jun 13;7:3424. doi: 10.1038/s41598-017-03427-z (PMC5469758; doi:10.1038/s41598-017-03427-z)
Supplement: Supplementary file 1 — Supplementary Information [file 41598_2017_3427_MOESM1_ESM.pdf]

SUPPLEMENTARY INFORMATION

Identification of an *in vivo* orally active dual-binding protein-protein interaction inhibitor targeting TNFα through combined *in silico/in vitro/in vivo* screening

Hadley Mouhsine, Hélène Guillemain, Gabriel Moreau, Najla Fourati, Chouki Zerrouki, Bruno Baron, Lucille Desallais, Patrick Gizzi, Nesrine Ben Nasr, Julie Perrier, Rojo Ratsimandresy, Jean-Louis Spadoni, Hervé Do, Patrick England, Matthieu Montes, Jean-François Zagury

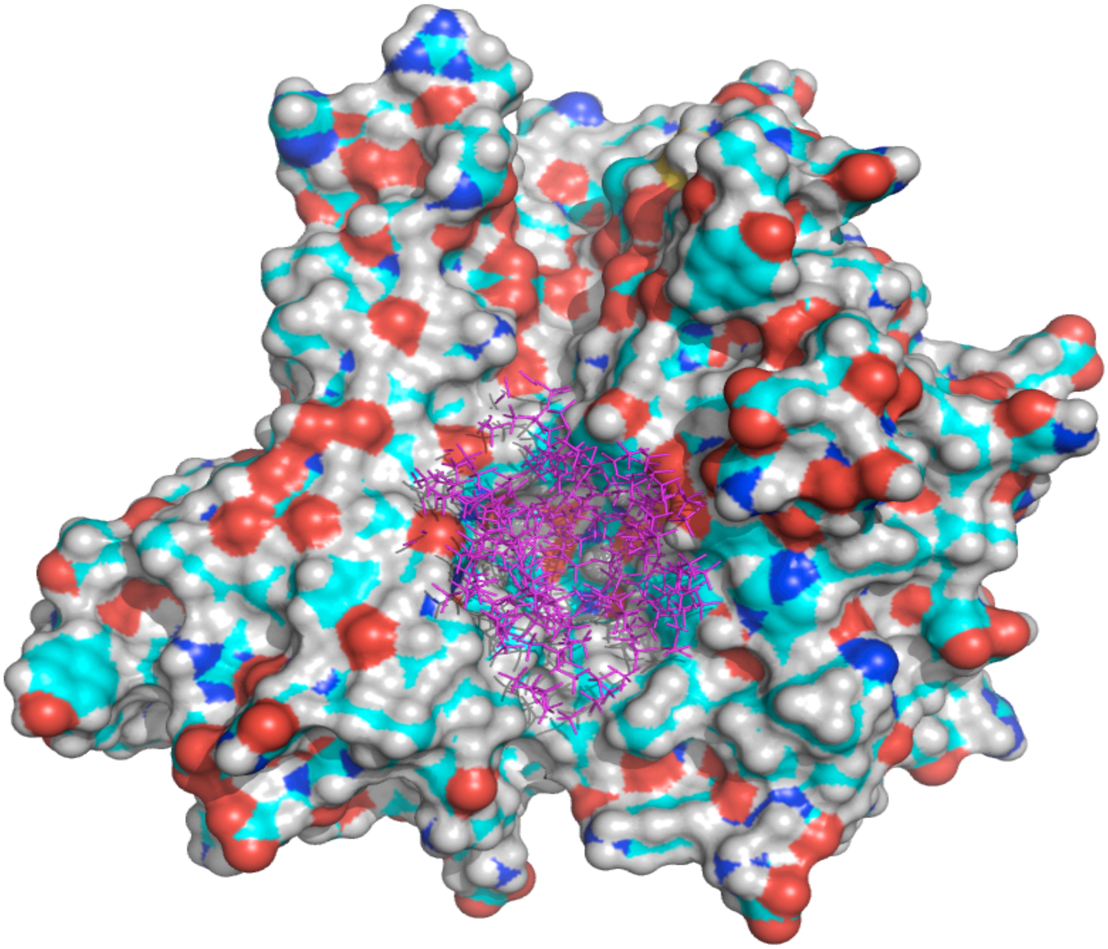

Figure S1. Surflex protomol (purple) used for the virtual screening experiments on the surface of the TNFa dimer (PDB ID: 2AZ5)

PRESTWICK CHEMICAL
A medicinal chemistry company

Prestwick Chemical SAS
Boulevard Gonthier d'Andernach
F-67400 Illkirch
Phone : +33 (0) 369 20 16 00
Fax : +33 (0) 369 20 16 17

CERTIFICATE OF ANALYSIS

Prestwick Number : PCI 10306

Name : N-[2-(4-Benzyl-piperazin-1-yl)-2-oxo-ethyl]-N-(3-chloro-2-methyl-phenyl)-benzenesulfonamide

Batch Number : MONTES-T0

Molecular Weight : 498.05

Molecular Formula : $C_{26}H_{28}ClN_3O_3S$

Chemists : n.a.

Quantity Shipped : n.a.

Date of Production : n.a.

Last Solvent : n.a.

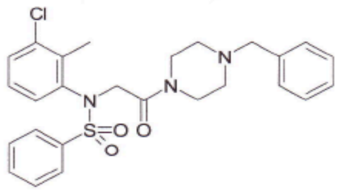

CHARACTERIZATION

Experience	Condition / Method	Result
Appearance		white powder
^{1}H NMR	300 MHz, DMSO-d_6	consistent - attached
LC/MS	gradient 5%-95% ACN/H_2O + 0.1% HCOOH (ESI$^+$)	consistent - attached<br>498.2 $[C_{26}H_{28}ClN_3O_3S+H]^+$ (m/z)

PURITY

Experience	Condition	Result
LC/MS	gradient 5%-95% ACN/H_2O + 0.1% HCOOH (ESI$^+$)	> 95%

SOLUBILITY

Water	DMSO	Benzyl alcohol	Propylene glycol
n.d.	+	n.d.	n.d.

Issued by J-M SIMON
Quality Assurance Manager

19/04/2013
Date

Approved by B. GIETHLEN
Chief Scientific Officer

19/04/2013
Date

Figure S2A. Analytics of compound 1.

Data file   : D:\DATA\MONTES-T0.D
Sample Name: MONTES-T0

Prestwick Chemical — A medicinal chemistry company

Prestwick Chemical SAS
Boulevard Gonthier d'Andernach
F-67400 Illkirch
Phone : +33 (0) 369 20 16 00
Fax : +33 (0) 369 20 16 17

Injection Date : Fri, 19. Apr. 2013
Sample Name : MONTES-T0
Acq Operator : EG

Column Description : Nucleodur
Column Length : 100 mm
Column Diameter : 4.6 mm
Particle size : 3.0 µm
Inj. Vol. : 5 µl

Solvent Description :
Solvent A : H_2O, 0.1% HCOOH
Solvent B : ACN, 0.1% HCOOH

Analysis Method : Gradient 5%-95% ACN/H2O

C8 DMSO

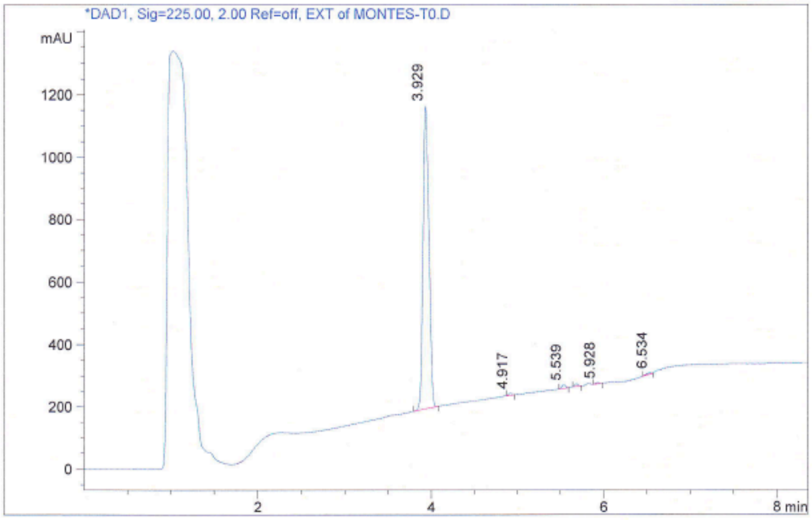

Signal 1: DAD1, Sig=225.00, 2.00 Ref=off, EXT

Peak #	RT [min]	Width [min]	Area	Area %
1	3.929	0.081	4725.713	97.994
2	4.917	0.044	17.833	0.370
3	5.539	0.045	31.097	0.645
4	5.684	0.046	21.580	0.447
5	5.928	0.050	10.161	0.211
6	6.534	0.072	16.058	0.333

Name SIMON
Date 19/04/13
Notebook QUA-E-115-2

*** End of Report ***

Figure S2B. Analytics of compound 1.

Display Report - Selected Window Selected Analysis

Analysis Name: MONTES-T0.D **Instrument:** Agilent 6310 Ion Trap **Print Date:** 19-Apr-13 08:57:58
Method: C8 DMSO.M **Operator:** Agilent **Acq. Date:** 19-Apr-13 08:45:58
Sample Name: MONTES-T0
Analysis Info:

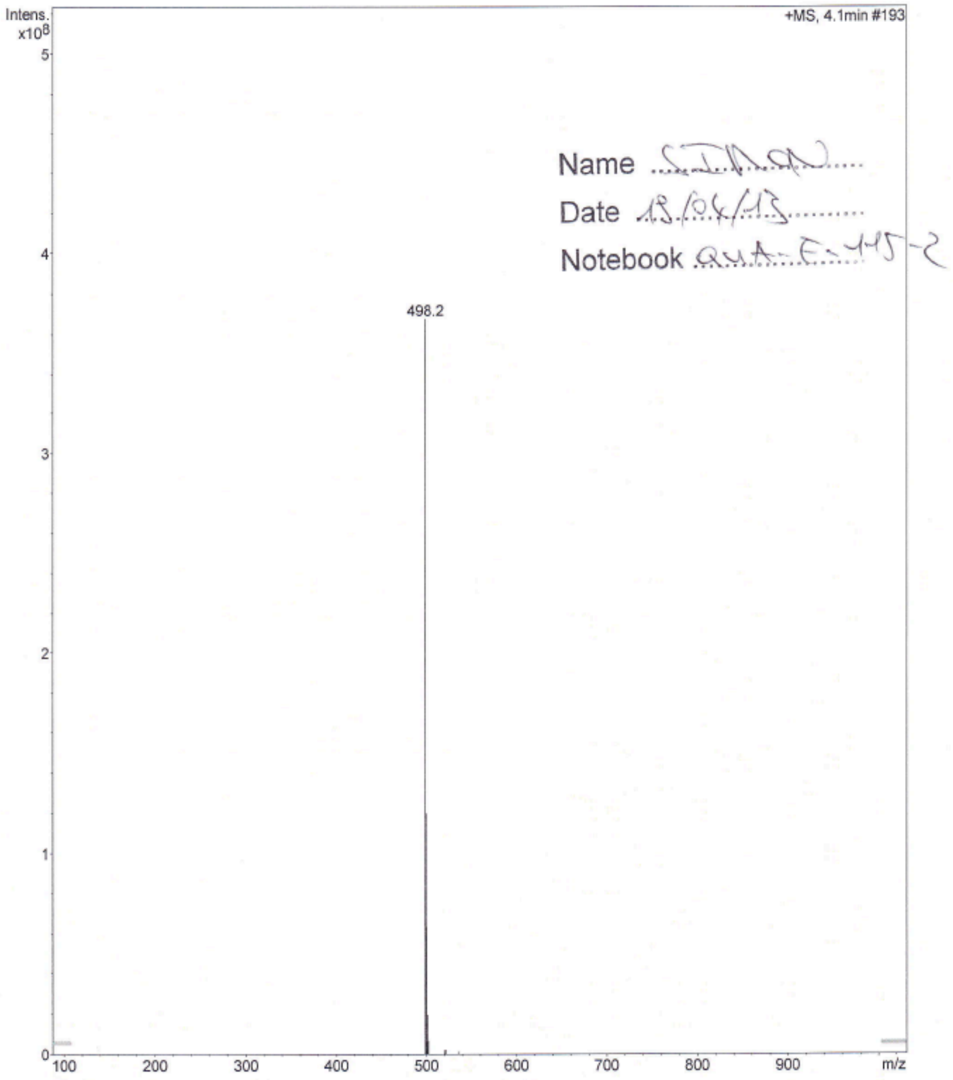

MSD Trap Report v 4 (A4-Opt2) Page 1 of 1 Agilent Technologies

Figure S2C. Analytics of compound 1.

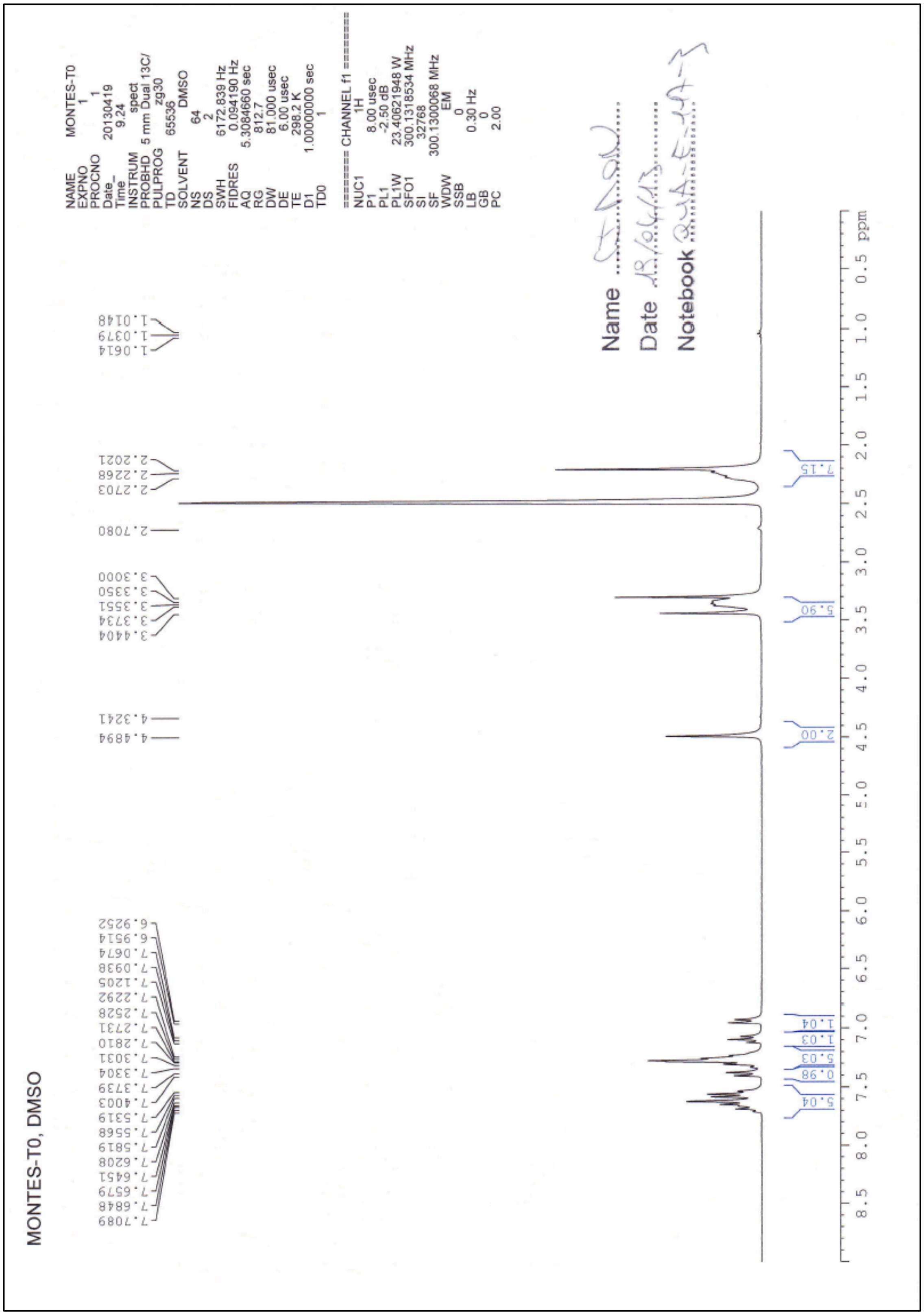

Figure S2D. Analytics of compound 1.

e

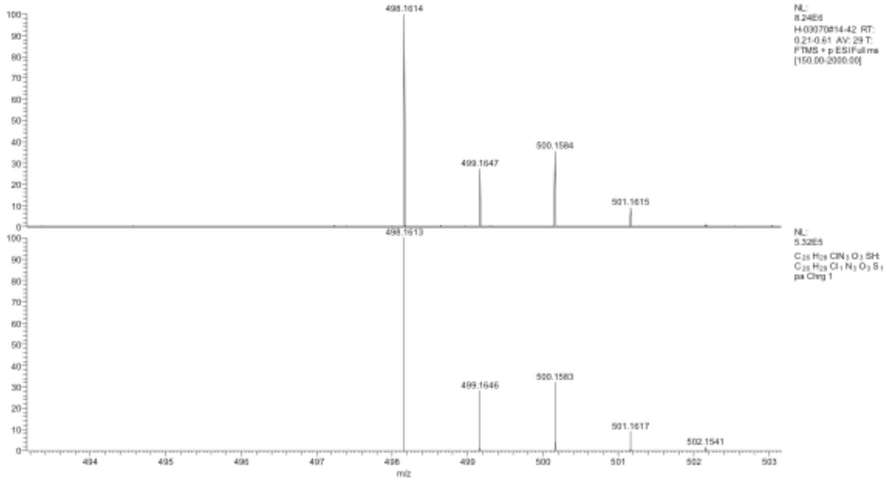

f

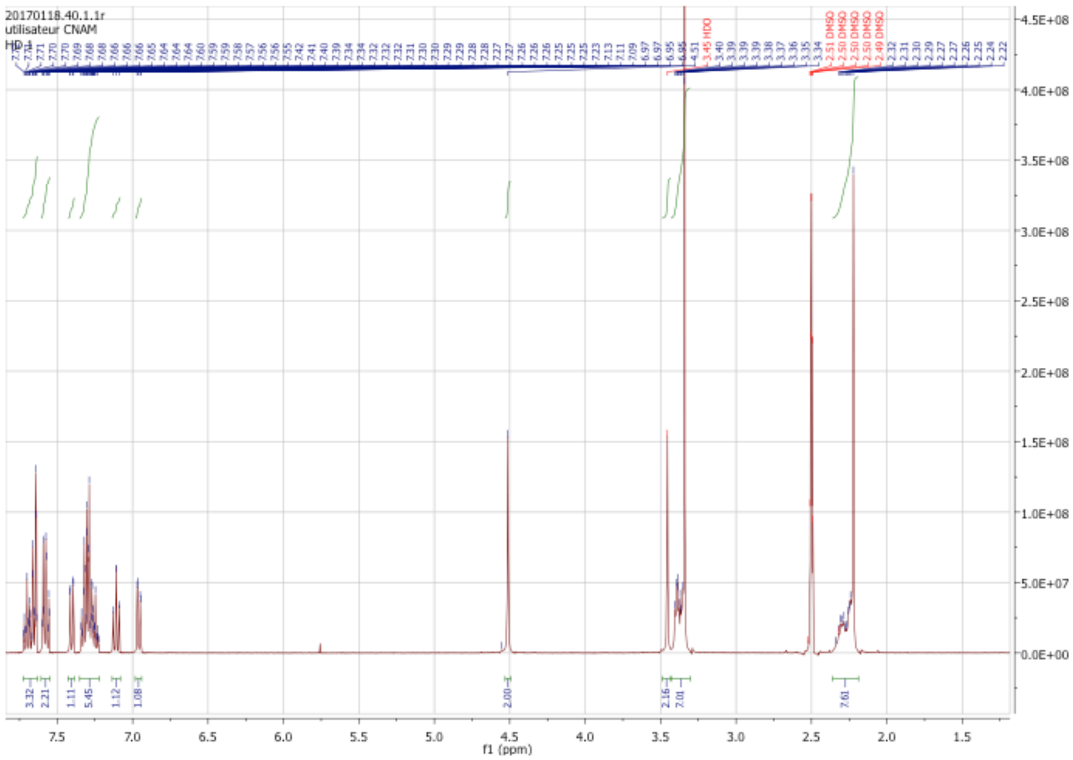

Figure S2. Analytics of compound 1.

g

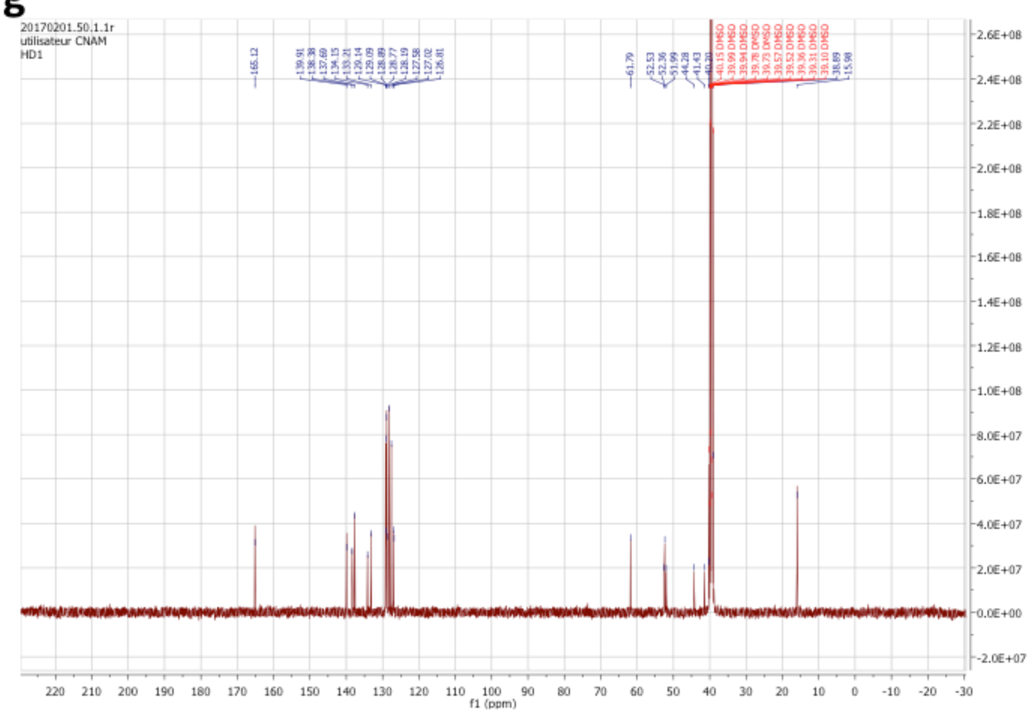

h

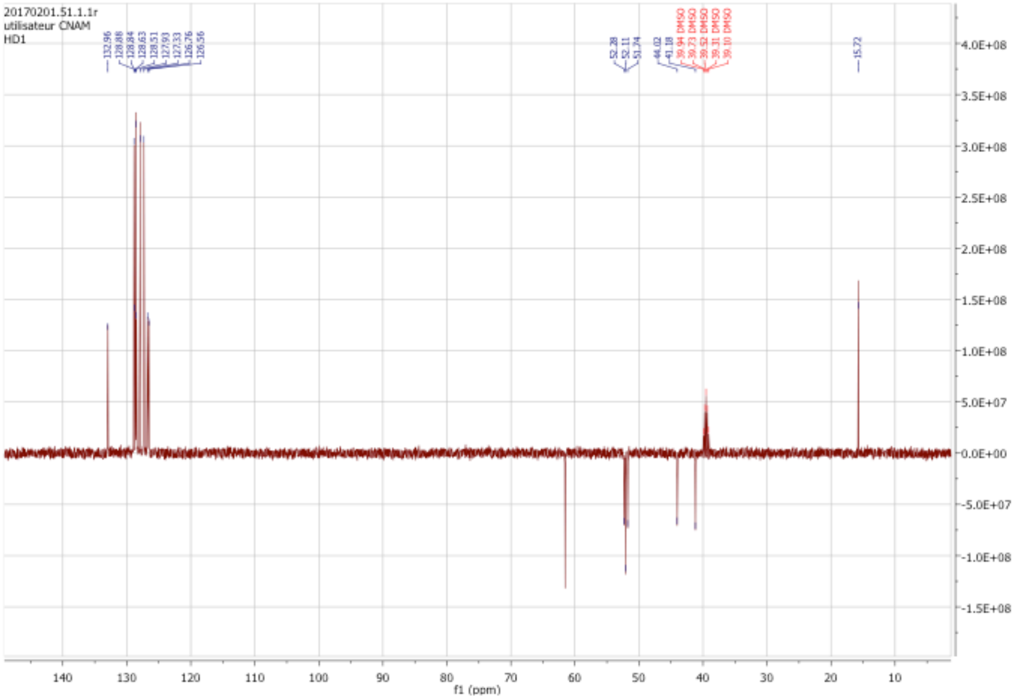

Figure S2. Analytics of compound 1.

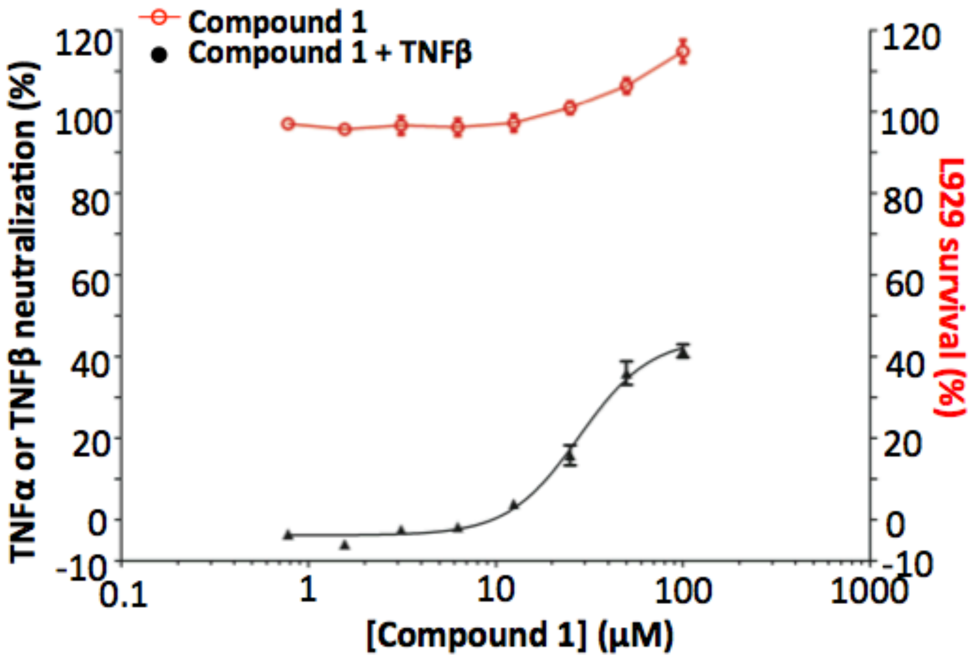

Figure S3. Neutralization of TNFβ and survival of L929 cells. Compound 1 inhibition of TNFβ induced apoptosis in L929 cell line. Data represent neutralization of TNFβ in presence of various concentrations of compound 1. IC_{50} = 28 μM

	**% Inhibition of Control Values**
**IKKα**	**3.2**
**IKKβ**	**10.3**
**IKKε**	**7.3**
**JNK1**	**-3**
**p38α kinase**	**1.5**
**p38γ kinase**	**-11**
**p38δ kinase**	**-1.8**
**Syk**	**-6.9**
**Caspase 3**	**-1.8**
**Caspase 8**	**-7.3**

Figure S4. ***In vitro*** **pharmacology of compound 1**. Compound 1 inhibition activity on kinases and caspases related to TNFα pathway. Compound 1 was tested at a concentration of 1 μM (CEREP Eurofins).
